# Dr. D. V. Stranahan

**Published:** 1892-08

**Authors:** 


Dr. D. V. Stranahan, of Warren, Pa., is dead at the age of forty-five years. He was the son of Dr. D. V. Stranahan and brother of Dr. J. L. Stranahan, of Erie, Pa. He graduated from the University of Buffalo in 1872, and had been in active practice until four years ago, when he was stricken with paralysis, since when he attended only to his office work. He enjoyed a large and lucrative practice, and was held in the highest esteem by his fellow-citizens.

